# Accuracy of Cervical Pedicle Screw Insertion With and Without a Navigation-Linked High-Speed Drill: A Retrospective Clinical Study

**DOI:** 10.7759/cureus.68558

**Published:** 2024-09-03

**Authors:** Kosei Ono, Sohei Murata, Mutsumi Matsushita, Yu Shimizu, Yusuke Nakamura, Taisuke Yabe, Hiromu Ito

**Affiliations:** 1 Department of Orthopedic Surgery, Kurashiki Central Hospital, Okayama, JPN

**Keywords:** neo classification, vertebral artery injury, high-speed drill, navigation, cervical pedicle screw

## Abstract

Introduction: Cervical pedicle screw (CPS) fixation provides high stability but poses a risk of nerve and vascular injury. Although useful for reducing CPS deviation rates, navigation systems cannot completely eliminate deviation. This study aimed to compare two methods for creating insertion paths, one using a navigation-linked high-speed drill (NAVI drill) and the other using conventional manual probing.

Methods: Our study comprised 104 patients with 509 CPSs at the C3-6 level who were treated at our institution between 2017 and 2023. CPS deviations were graded according to the Neo classification system, and the deviation direction (medial, lateral, cranial, or caudal) was assessed. Complications associated with CPS deviation were also investigated. We compared cases that used the NAVI drill (Group M) with those that used manual probing (Group N).

Results: Group M included 45 cases (252 screws), and Group N included 59 cases (257 screws). The CPS deviation rate was grade 1 or higher in 14.7% and 17.1% of cases in Groups M and N, respectively (p = 0.469). It was grade 2 or higher in 1.2% and 4.3% of cases in Groups M and N, respectively (p = 0.222). The medial, lateral, caudal, and cranial deviation direction rates were 56.8%, 2.7%, 40.5%, and 0% in Group M and 13.6%, 72.7%, 11.4%, and 2.3% in Group N, respectively (p < 0.001). In one case in Group N, a grade 3 lateral deviation resulted in vertebral artery injury (VAI).

Conclusions: The use of the NAVI drill was associated with a slightly lower, albeit insignificant, CPS deviation rate. However, it significantly lowered the proportion of lateral deviations. Therefore, the NAVI drill is a useful tool for preventing VAI.

## Introduction

Cervical pedicle screw (CPS) fixation for the middle and lower cervical spine was first reported by Abumi et al. in 1994 [1]. CPSs have high pullout strength and offer greater stability than other anchors [2], making them useful for the fixation of unstable fractures and in corrective cervical surgeries. They are also a good option for patients with bone fragility due to osteoporosis. Unlike hooks or wires, CPSs can be easily used in decompression procedures such as cervical laminectomy and laminoplasty. Despite their many advantages, there is a risk of serious complications associated with their use [1].

Accurate placement of CPSs is challenging owing to the anatomical features of the cervical pedicle. The medial aspect of the pedicle houses the spinal cord, nerve roots border the cranial and caudal aspects, and the vertebral artery lies on the lateral side. CPS deviation poses a risk of vascular and neural injuries. In particular, deviation toward the lateral side can cause vertebral artery injuries (VAIs) and consequent cerebellar or brainstem infarction, which is a severe life-threatening complication [3].

CPSs are inserted freehand or using fluoroscopy-guided techniques. Navigation technology facilitates real-time visualization of anatomical structures during surgery and is a useful tool for improving CPS placement accuracy. However, it does not completely eliminate screw deviations [4-14]. Our facility uses a navigation system based on intraoperative three-dimensional (3D) C-arm fluoroscopy for CPS insertion. Initially, manual probes and drills linked to the navigation system were used to create entry paths. Subsequently, to improve the accuracy of the entry path creation, we introduced the use of a navigation-linked high-speed drill (NAVI drill). This study aimed to investigate whether NAVI drill use improved the accuracy of CPS insertion procedures.

## Materials and methods

This study was approved by our institutional review board (Kurashiki Central Hospital, Medical Ethics Committee, approval number: 4407). Owing to its retrospective nature, the need for informed consent was waived. The patients were provided with an opt-out option.

One hundred four cases (comprising 509 screws), between January 2017 and July 2023, in which CPS was used were included. Non-CPS screws such as lateral mass screws were excluded. Patient demographics, including age, sex, body mass index (BMI), diagnosis, number of fixed vertebral levels, and use of the NAVI drill, were evaluated.

Immediately after surgery, computed tomography (CT) was performed to assess screw deviation using the Neo classification system, which grades deviations from 0 to 3 [6]. Screw deviation was evaluated in four directions: medial, lateral, cranial, and caudal. Additionally, the complications associated with screw deviation immediately after surgery were investigated. The complications included muscle weakness (a decrease in the manual muscle score of ≥1) but excluded late-onset C5 paralysis and symptomatic VAIs. The cases were divided into two groups for comparative analysis: Group M (NAVI drill used) and Group N (NAVI drill not used).

Surgical technique

The patient was placed in the prone position. A midline longitudinal skin incision was made over the posterior aspect of the cervical spine to expose the laminae. A reference frame was placed on the spinous process. Intraoperative 3D imaging was performed using a C-arm fluoroscopy system (Arcadis Orbic 3D C-arm, Siemens, Erlangen, Germany). A navigation system (StealthStation S7; Medtronic, Minneapolis, MN) based on the 3D images was used during this procedure.

In Group M, entry paths were created using the NAVI drill linked to a navigation system (Midas Rex MR8; Medtronic). In Group N, entry paths were manually created by pushing probes linked to the navigation system. Subsequent tapping and screw insertion were performed in both groups.

Statistical analysis

Data are presented as mean ± standard deviation unless otherwise specified. Differences between Groups M and N were evaluated using the Fisher exact test and Mann-Whitney U test for categorical and continuous variables, respectively. Statistical significance was set at p < 0.05. EZR version 1.67 (Saitama Medical Center, Jichi Medical University, Saitama, Japan) was used for all analyses.

## Results

Among the 104 patients (71 males and 33 females) in our study, the average age was 69.9 ± 12.1 years, and the average BMI was 22.8 ± 3.9 kg/m^2^ (Table 1). The diagnoses included cervical spondylotic myelopathy (32 cases), ossification of the posterior longitudinal ligament (23 cases), fracture or dislocation (33 cases), tumor (15 cases), and infection (one case). The average number of fixed vertebral levels was 4.3 ± 2.2.

**Table 1:** Demographic data of the 104 patients BMI, body mass index; CSM, cervical spondylotic myelopathy; OPLL, ossification of the posterior longitudinal ligament; NAVI drill, high-speed drill linked to a navigation system Data are presented as mean ± standard deviation unless otherwise specified.

Variable	Value
Age (years)	69.9 ± 12.1
Sex	
Male	71
Female	33
BMI (kg/m^2^)	22.8 ± 3.9
Diagnosis	
CSM	32
OPLL	23
Fracture	33
Tumor	15
Infection	1
Number of fixed vertebral levels	4.3 ± 2.2
NAVI drill	
Yes	45 (Group M)
No	59 (Group N)

The overall screw deviations are shown in Table 2. The deviation rate was grade 1 or higher in 15.9% of cases and grade 2 or higher in 2.2% of cases.

**Table 2:** Neo classification

Grade	Number of screws (N = 509)
1	70
2	10
3	1

The NAVI drill was used in 45 cases with 252 screws (Group M), and manual probes were used in 59 cases with 357 screws (Group N). No significant differences between the Groups M and N were observed in the demographic data (Table 3).

**Table 3:** Demographic data: Group M versus Group N BMI, body mass index; CSM, cervical spondylotic myelopathy; OPLL, ossification of the posterior longitudinal ligament Data are presented as mean ± standard deviation unless otherwise specified. Differences between Groups M and N were evaluated using the Fisher exact test and Mann-Whitney U test for categorical and continuous variables, respectively. Statistical significance was set at p < 0.05.

Variable	Group M	Group N	p
Number	45 (252 screws)	59 (257 screws)	
Age (years)	70.6 ± 12.9	69.3 ± 11.6	0.446
Sex			
Male	32	39	0.673
Female	13	20	
BMI (kg/m^2^)	23.2 ± 3.6	22.4 ± 4.2	0.175
Diagnosis			
CSM	16	16	0.409
OPLL	12	11	
Fracture	13	20	
Tumor	4	11	
Infection	0	1	
Number of fixed vertebral levels	4.5 ± 1.9	4.2 ± 2.4	0.455

Table 4 shows the Neo classification grades for Groups M and N. The CPS deviation rate was grade 1 or higher in 14.7% and 17.1% of cases in Groups M and N, respectively (p = 0.469). It was grade 2 or higher in 1.2% and 4.3% of cases in Groups M and N, respectively (p = 0.222). Although there was a slight tendency toward a lower deviation in Group M, the difference between the groups was not significant.

**Table 4:** Neo classification: Group M versus Group N

Variable	Group M	Group N
Total number of screws	252	257
Grade 1	34	36
Grade 2	3	7
Grade 3	0	1

Further, we compared the deviation direction rates between Groups M and N (Table 5). The deviations in Group M were mostly in the medial and caudal directions, whereas those in Group N were mainly in the lateral direction (p < 0.001).

**Table 5:** Deviation direction: Group M versus Group N Differences between Groups M and N were evaluated using the Fisher exact test and Mann-Whitney U test for categorical and continuous variables, respectively. Statistical significance was set at p < 0.05.

Deviation screws	Medial	Lateral	Cranial	Caudal
Group M (37 screws)	21 (56.8%)	1 (2.7%)	0 (0%)	15 (40.5%)
Group N (44 screws)	6 (13.6%)	32 (72.7%)	1 (2.3%)	5 (11.4%)
p	<0.001			

**Figure 1:**
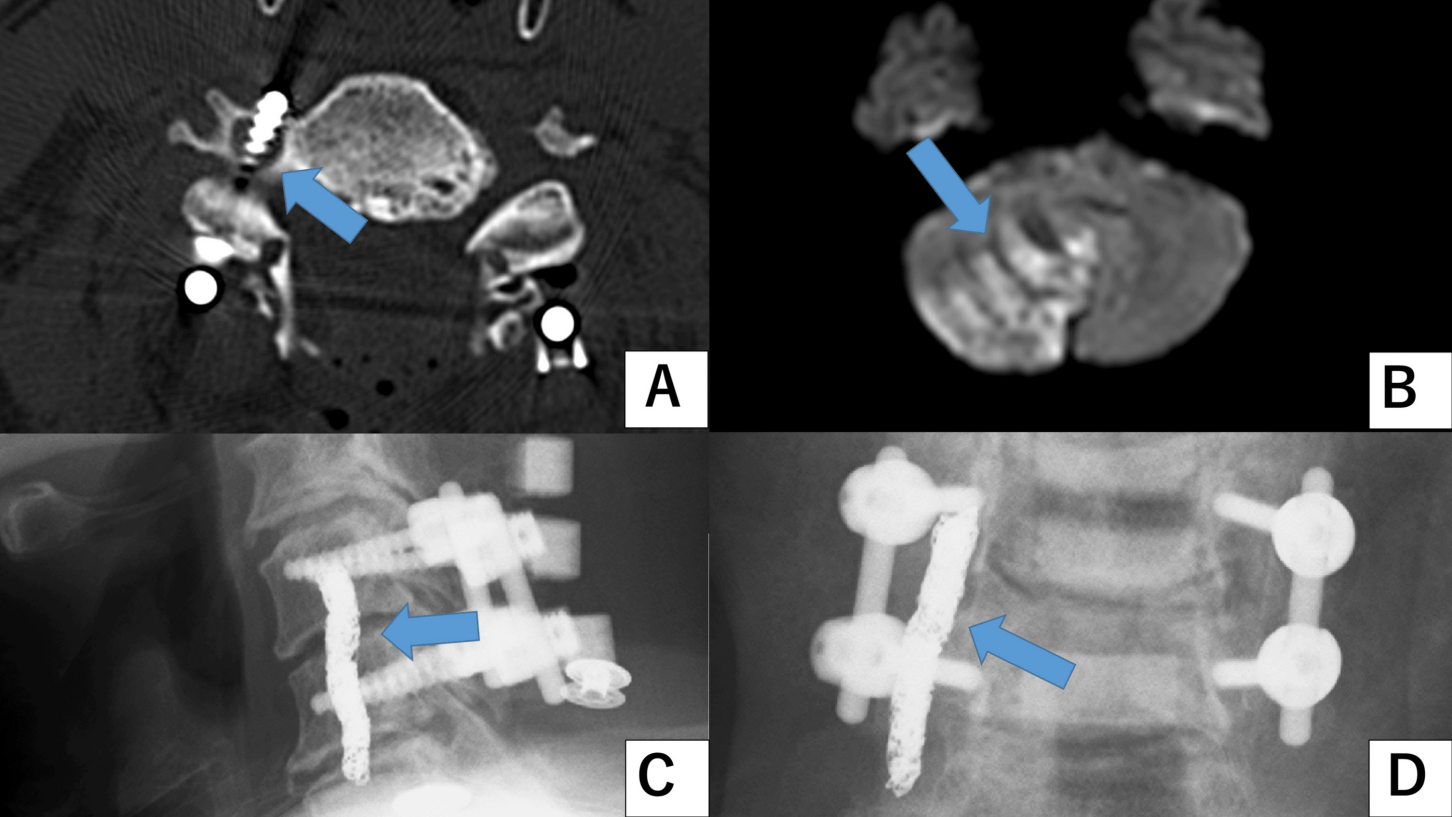
Imaging results (Group N, lateral, grade 3 deviation) A: Computed tomography image of the dislodged screw. B: Magnetic resonance image of the cerebellar infarction. C and D: Post-embolization X-ray images.

In one Group N case with a lateral grade 3 deviation, VAI occurred, which led to cerebellar infarction. Embolization was performed after consultation with the neurointerventional team. Figure 1 shows the CT image of the dislodged screw, magnetic resonance image of the cerebellar infarction, and post-embolization X-ray image.

## Discussion

The introduction of pedicle screws by Roy-Camille et al. in 1986 led to significant improvements in the outcomes of thoracolumbar spine reconstructive surgery [15]. However, for the reconstruction of the cervical spine, advancements in internal fixation techniques have been slower owing to anatomical constraints such as smaller bone size and proximity to critical structures (the spinal cord, nerve roots, and vertebral artery). Following the report by Abumi et al. in 1994, clinical CPS procedures were initially performed freehand or using fluoroscopy [1]. CPS procedures became more universally applicable following the development of surgical support tools, such as navigation systems and spinal robots, ensuring safety and precision irrespective of the surgeon's experience [13].

Pedicle screws provide high fixation stability owing to their insertion into thick cortical bone, making them suitable for corrective procedures (e.g., kyphosis correction) and conditions (e.g., highly unstable cervical spine fractures) requiring reliable fixation over a short range [2,9]. These devices are also considered appropriate in cases of bone fragility due to conditions such as osteoporosis and rheumatoid arthritis, whereas other fixation methods may pose stability concerns. Unlike wire or hook systems, CPSs allow for combined posterior decompression because they do not rely solely on the lamina or spinous process for fixation. However, their proximity to neural and vascular structures is potentially harmful, necessitating careful attention during use.

Damage to the vertebral artery during pedicle screw insertion increases the risk of cerebral infarction. Even in cases in which the vertebral artery is not directly damaged during surgery, significant lateral movement of the pedicle screw into the transverse foramen can cause delayed cerebral infarctions, warranting caution [3]. In our study, a grade 3 lateral deviation in Group N resulted in VAI and cerebellar infarction, necessitating embolization. Fortunately, there were no recurrent strokes post-embolization, and the patient's progress was favorable. Given the considerable anatomical variations of the vertebral artery, preoperative assessment using contrast-enhanced CT or magnetic resonance angiography tailored for individual cases is necessary.

The reported accuracy of CPS insertion without navigation varies from 6.7% to 31.6% [4-12]. Identifying precise insertion points without landmarks remains challenging, especially in cases of degeneration or trauma affecting anatomical landmarks. Despite the use of CT-based navigation, errors due to patient positioning or cervical alignment still result in deviation rates of 1.2%-18.7% [10-14], questioning the absolute safety of this method. The reported rates of nerve root injury and VAI range from 0.2% to 0.8% and 0.2% to 0.5%, respectively [4,9,16]. The thin and short anatomy of the cervical pedicle, lack of clear landmarks, and proximity of the cervical pedicle to critical structures necessitate a thorough understanding of cervical spinal anatomy for safe screw insertion.

Although the deviation rate in this study was not significantly lower when the NAVI drill was used, there was a slight trend toward a lower deviation rate. The proportion of lateral deviations was lower in the presence versus absence of the NAVI drill, possibly because the NAVI drill creates insertion paths with less manual force than probes, thereby reducing the likelihood of lateral CPS deviation caused by vertebral body rotation during path creation. The reported angle from the vertical direction of the cervical pedicle is 30°-62°, with an average of 46° [17,18]. When creating an access path via manual probe insertion, the angle tends to increase further owing to vertebral body rotation, thereby increasing the risk of lateral deviation. The strong drilling force of the NAVI drill may also reduce the likelihood of deviation toward the softer lateral cortex after being deflected by the hard medial cortex [19].

There is considerable space between the inner wall of the pedicle and the spinal cord, with the vertebral artery proximate to the outer wall, necessitating caution against lateral deviation. Surgeons consciously aim to insert screws slightly medial to the center of the pedicle to minimize lateral deviation; this may account for the relatively high percentage of medial deviations in Group M. Fortunately, most deviations were grade 1, and no nerve damage was observed.

This study has some limitations. First, because it was retrospective, there was a potential for selection bias. The use of the NAVI drill may have been suitable for narrow pedicles previously considered unacceptable for CPS fixation; this could have contributed to the lack of significant differences in the deviation rates between Groups M and N. Second, the relatively small sample size could have affected the generalizability and statistical power of the findings. Third, the clinical scores of the patients were not individually evaluated, which perhaps limited a comprehensive understanding of the impact of the intervention on patient outcomes. Fourth, owing to the lack of routine postoperative contrast-enhanced CT or head magnetic resonance imaging, asymptomatic VAIs or cerebral infarctions may have been overlooked. Furthermore, sensory deficits were not assessed, potentially leading to an underestimation of the number of nerve injuries.

## Conclusions

The use of the NAVI drill did not significantly affect the CPS deviation rate; however, a tendency toward a lower deviation rate was observed, specifically a significantly lower deviation toward the lateral side. The use of the NAVI drill may reduce the frequency of lateral deviation, as well as the risk of complications such as VAI and cerebellar infarction. However, medial and caudal deviations need to be prevented as well.
